# γδ T Cells in HIV Disease: Past, Present, and Future

**DOI:** 10.3389/fimmu.2014.00687

**Published:** 2015-01-30

**Authors:** C. David Pauza, Bhawna Poonia, Haishan Li, Cristiana Cairo, Suchita Chaudhry

**Affiliations:** ^1^Institute of Human Virology and Department of Medicine, University of Maryland School of Medicine, Baltimore, MD, USA

**Keywords:** gamma delta T cell, HIV, Vdelta1 gamma delta T cells, Vgamma9Vdelta2 T cells, immunotherapy

## Abstract

Human immunodeficiency virus (HIV) type 1 dysregulates γδ T cells as part of an immune evasion mechanism. Nearly three decades of research defined the effects of HIV on γδ T cells and how this impacts disease. With highly effective antiretroviral therapy providing virus suppression and longer survival, we expected a return to normal for γδ T cells. This is not the case. Even in patients with CD4 T cell reconstitution, normal γδ T cell levels and function are not recovered. The durable damage to Vδ2 T cells is paralleled by defects in NK, CD8 T cells, and dendritic cells. Whether these consequences of HIV stem from similar or distinct mechanisms are not known and effective means for recovering the full range of cellular immunity have not been discovered. These unanswered questions receive too little attention in the overall program of efforts to cure HIV this disease. Approved drugs capable of increasing Vδ2 T cell function are being tested in clinical trials for cancer and hold promise for restoring normal function in patients with HIV disease. The impetus for conducting clinical trials will come from understanding the significance of γδ T cells in HIV disease and what might be gained from targeted immunotherapy. This review traces the history and current progress of AIDS-related research on γδ T cells. We emphasize the damage to γδ T cells that persists despite effective virus suppression. These chronic immune deficits may be linked to the comorbidities of AIDS (cancer, cardiovascular disease, metabolic disease, and others) and will hinder efforts to eradicate HIV by cytotoxic T or NK cell killing. Here, we focus on one subset of T cells that may be critical in the pathogenesis of HIV and an attractive target for new immune-based therapies.

## Original Studies on HIV and γδ T Cells

Human immunodeficiency virus (HIV) is an aggressive, lymphotropic virus known for CD4 depletion and immune suppression. In addition to killing CD4 T cells, HIV affects several other lymphocyte subsets (1) and impairs both acquired and innate immunity (2). We focus on HIV damage to γδ T cells and how this is related to acute or chronic disease. The two major subsets of human γδ T cells (designated Vδ1 or Vδ2) are both altered after HIV infection. Early reports that Vδ1 T cells were increased (3) and that the normal ratio of Vδ2:Vδ1 cells was inverted (4) identified an important and reproducible effect of HIV on these CD4-negative T cells. The increased levels of Vδ1 cells suggested they may be involved in antiviral immunity (5) and parallels were drawn between the expansion of Vδ1 cells and similar increases in CD8+ T cells (5) that contribute to the inverted CD4:CD8 T cell ratio (6). Non-human primate studies showed that Vδ1 cell expansion is an indirect consequence of viral infection and reflects increased translocation of stimulatory bacterial products across the gut epithelium (7). The Vδ1 cells may have antiviral functions through killing of infected cells using the NKp30 (8) or NKG2C recognition receptors (9). Killing of uninfected CD4 T cells by Vδ1 cells may also be a mechanism for HIV disease (10). Whether Vδ1 T cells accelerate or slow HIV progression has not been resolved.

In contrast to Vδ1 cells, the Vδ2 subset is uniformly depleted in HIV disease with the greatest declines seen among patients with high viremia (11). These early studies [before the introduction of combination antiretroviral therapy (ART)] involved patients who were untreated or received single drug therapy where viremia was reduced 10–50-fold but never suppressed fully. The lowest levels of Vδ2 cells were found in patients with opportunistic infections or CD4 T cells < 200 cell/mm^3^ of blood (12, 13). These individuals frequently had no detectable cells bearing the phosphoantigen-responsive Vγ9 chain (14). Importantly, decreases in Vδ2 cells and inversion of the Vδ2:Vδ1 cell ratio were early events in HIV disease that occurred while CD4 cell counts and their CD4:CD8 T cell ratio were still in the normal ranges.

Molecular analysis of γ and δ T cell receptor (TCR) chain usage showed that HIV-driven expansion of Vδ1 cells did not select for specific Vδ chain rearrangements or individual Vγ chains (15). Sequencing studies to describe the TCR repertoire confirmed that the population of Vδ1 chains was similar in donors with or without HIV infection and that HIV-driven Vδ1 cell expansion was not similar to antigen selection in αβ T cells (16) but more likely was a polyclonal or super-antigen response. In contrast to the case for Vδ1 cells, there was strong evidence for selective Vδ2 cell depletion based on TCR structure. Flow cytometry studies documented the specific loss of Vγ9 (also called Vγ2) expressing cells, a chain more frequently associated with Vδ2 than Vδ1 in healthy controls. Finding that the relative proportion of Vγ9 expressing cells was decreased in HIV patients, being lower in both blood and bronchoalveolar lavage specimens despite having increased Vδ1 cells (17), encouraged a closer look at the Vγ9Vδ2 subset. The importance of Vγ9Vδ2 T cell depletion and the relationship between depletion and TCR became more clear as other groups defined antigens for these unusual cells.

## Finding the Antigens for Vδ2 T Cells

Major activities of Vγ9Vδ2 T cells include a strong response to Mycobacterium-infected human PBMC, even though these cells do not react to purified mycobacterial 65-kd heat shock protein (18). The Vγ9Vδ2 T cells respond to Mycobacteria-pulsed cells or antigens found on the human MOLT-4 lymphoma cell line (19), which was confusing in the context of the model for MHC-restriction that explained αβ T cell recognition of peptide antigens. Recognition of either Mycobacterium-pulsed cells or lymphoma cell lines required a specific rearrangement between the variable γ9 (Vγ9) and joining P (JP) segments; constant region 1 (C1) was incorporated by mRNA splicing to form Vγ9JPC1 chains that combine with a Vδ2DJC chains to form functional receptors (20, 21). In an alternate nomenclature, the V-J rearrangement is designated Vγ2Jγ1.2; the nomenclature is consistent for δ chains. This combination of specifically rearranged Vγ9 with Vδ2 chains endowed the capacity for recognizing mycobacterial antigens (22, 23). Efforts to characterize stimulatory molecules showed first they were phosphorylated (24) and later that they were prenyl pyrophosphate intermediates of sterol synthesis in mammalian cells (25). A variety of natural and non-natural molecules were tested for Vγ9Vδ2 T cell stimulation to define the essential antigenic structure (26) within a group of chemicals now known as phosphoantigens. The natural abundance of mammalian phosphoantigens, being made in every living cell, in addition to structurally similar but often more potent compounds produced by bacteria, protists, plants, or fungi, serves to inundate human physiology with stimulators of Vγ9Vδ2 T cells. These compounds select for and amplify the Vγ9JPVδ2 cell subset during early life as was described in a remarkable paper from Michael Brenner’s group (27).

In children, a fetal Vγ9 chain repertoire is replaced slowly with one dominated by the Vγ9JγP rearrangement. Under continuous positive selection, the Vγ9Vδ2 cell count rises and phosphoantigen-responsive cells are increasingly found in blood as central or effector memory types with declining proportions of naïve cells. Healthy adults maintain a diverse but highly redundant repertoire such that 1 in 40 circulating memory T cells is a phosphoantigen-responsive Vγ9Vδ2 T cell. Clearly, the Vγ9JPVδ2 cell dominates T cell memory in healthy adults. Baseline levels of Vγ9Vδ2 T cells differ by two to fourfold between white European-origin or Asian-origin (high) and African-origin (low) peoples but repertoire complexity is similar among these groups and *in vitro* responses to phosphoantigen are also similar (28). Positive selection and amplification of Vγ9JPVδ2 T cells is ubiquitous in man and present in most non-human primate species studied so far, but is not present in lower mammals including rodents that lack both a gamma chain gene similar to Vγ9 and butyrophilin 3A1 that is also required for phosphoantigen responses (29–34).

## Specific Destruction of Antigen-Specific Vδ2 T Cells in HIV Disease

Two important papers in 1996 and 1997 helped to bridge HIV studies with the emerging understanding of phosphoantigens and their importance to γδ T cell biology. Gougeon’s group confirmed earlier studies on Vδ2 cell depletion in HIV patients and reported a disease-associated “functional anergy” measured by lack of proliferation or cytokine responses after stimulation with mycobacterial antigens (35). These authors studied the junctional diversity of Vγ9Vδ2 TCR chains expressed in HIV+ individuals and reported that the Vδ2 cell chain repertoire remained diverse. They also noted there were no differences in spontaneous apoptosis between HIV patients or uninfected control donors after *in vitro* phosphoantigen stimulation. A second group led by Malkovsky confirmed the functional anergy in Vδ2 T cells from HIV patients by documenting decreased responses to phosphoantigen or to the prototypical cell target Daudi B cell (36). Both groups noted that Vδ2 T cells were reduced but not eliminated in HIV disease, and were substantially deficient in their response to phosphoantigen due to anergy that may have resulted from inappropriate activation *in vivo*. A smaller study of HIV+ individuals noted differences from controls regarding γδ T cell responses to *Salmonella typhimurium* or *Candida albicans*, and reported that Vδ2 cell responses to Mycobacteria remained intact only in patients with >500 CD4 T cells/mm^3^ (37). Further, Vδ2 cells were depleted from blood but increased in liver from both HIV patients and HIV-negative patients who had disseminated *Mycobacterium avium* complex (38). Vδ1 cells were increased in tissue sites among HIV patients, notably liver (39) or bone marrow (40). The pattern of changes among γδ T cells for both Vδ2 and Vδ1 cells was a distinguishing feature of HIV disease.

## Milestone Achievements from Early Studies on γδ T Cells in HIV Disease

By 1997, there was a basic understanding of HIV infection and its impact on γδ T cells. Four major concepts had emerged: (1) Inversion of the Vδ2:Vδ1 cell ratio was an early event, occurring prior to inversion of the CD4:CD8 T cell ratio. (2) Vδ1 cells are increased in patients with HIV. (3) The Vδ2 cell depletion was accompanied by decreased responsiveness to phosphoantigens or tumor cells. (4) Loss of Vδ2 cells was greatest in patients with low CD4+ T cells, high viremia, opportunistic infections and late stage disease (AIDS). Consequently, HIV-mediated changes in γδ T cells appear to be part of the mechanism for evading antiviral immunity and establishing persistent infection with chronic disease. Persistent infection is essential for viruses like HIV that are transmitted with relatively low efficiency and require direct person-to-person contact. These studies highlighted the need to understand mechanisms for γδ T cell dysregulation, define impacts of these changes on immunity to HIV and look more broadly at “unintended consequences” of the viral immune evasion strategy.

## Mechanisms for Dysregulating γδ T Cells

Model studies in non-human primates have helped to explain some of the γδ T cell changes during disease. Because rodents lack the TCR sequences needed for phosphoantigen recognition, studies on Vγ9Vδ2 T cells have been restricted to human beings and non-human primates. A recent genome mining study revealed that functional genes for Vγ9Vδ2 and butyrophilin 3A1 were actually present in a few other placental mammals including alpaca, sloth, bottlenose dolphin, killer whale, horse, and armadillo (41). Most of these species are unfamiliar experimental systems with the exception of armadillo that has been used for research on *Mycobacterium leprae*, but we can expect humans and non-human primate models to dominate this field for the foreseeable future.

When peripheral blood Vγ9Vδ2 T cells were isolated from uninfected rhesus monkeys (naïve to viral antigens) they were directly cytotoxic for SIV-infected target cells (42). There were rapid increases in blood Vγ9Vδ2 T cell counts and higher expression of activation markers within a few days after SIV infection of rhesus monkeys even though these cells were already showing decreased proliferation responds *in vitro* (43). The Vγ9Vδ2 T cell expansion seen early after SIV infection of macaques was brief and was followed by a rapid decline in cell count and function. This animal model recapitulates the decline in Vγ9Vδ2 T cells and inversion of the Vδ2:Vδ1 cell ratio. Similarly, Vγ9Vδ2 cell lines or clones from HIV-negative donors recognized and killed HIV-infected cells (44). It was also known that healthy Vγ9Vδ2 cells produced large amounts of interferon-γ, tumor necrosis factor-α, and chemokines RANTES or MIP-1β (45) that were associated with antiviral immunity. Normally, the circulating Vγ9Vδ2 cells (mostly memory phenotype) have preformed cytoplasmic vesicles containing RANTES that are released immediately upon phosphoantigen stimulation or after contact with target cells (46). The release of RANTES and other chemokines suppressed HIV replication *in vitro* by blocking co-receptors for virus entry (47, 48). The CCR5 receptor is highly expressed on Vδ2 cells (49) meaning RANTES release would attract even more Vγ9Vδ2 cells able to release additional chemokine that would block HIV entry, kill already infected cells through direct cytotoxicity, or mediate antibody-dependent cellular cytotoxicity through cell surface Fc γ receptors (50). Circulating Vγ9Vδ2 cells also activate innate and acquired immunity through the release of pro-inflammatory IFN-γ and TNF-α or regulatory cytokines (51).

While it seemed clear that γδ T cell dysregulation is part of HIV immune evasion, the precise impact on pathogen immunity was less clear. Little was known about how Vδ1 expansion or Vδ2 T cell depletion affect normal immunity and whether these are important aspects of viral immune suppression linked to progressing disease. Brenchley’s group (7) reported that Vδ1 cell expansion in SIV-infected rhesus monkeys was related to pathologic changes in the intestinal epithelium that increased bacterial translocation causing higher levels of bacterial products in circulation that would stimulate Vδ1 T cells. The Vδ1 cells were also increased by influenza vaccination in HIV patients but only if the vaccine contained MF-59 adjuvant (52). Thus, Vδ1 cells are responsive to stimulation in HIV patients and expansion could be linked to bacterial translocation or reflect the normal responses to *Candida albicans* (53) and other common intercurrent infections in HIV patients.

Like Vδ1, phosphoantigen-responsive Vδ2 cells increase soon after infection as was documented in the SIV infection of macaques (43) but then are depleted and often extinguished. When cloned Vδ2 T cells are stimulated by anti-CD3 monoclonal antibody plus IL-2 they frequently die due to apoptosis, yet anti-CD3 stimulation plus feeder cells in the absence of exogenous IL-2 leads to proliferation (54). Thus, stimulation conditions impact outcome. It remains difficult to extrapolate *in vitro* conditions into explanations for *in vivo* outcomes and the mechanism for HIV-mediated depletion are still unclear. Laboratories working with Vδ2 T cells, including our groups, know that phosphoantigen plus IL-2 stimulation of human or macaque PBMC results in rapid cell death followed by outgrowth of a surviving population that peaks around 10–14 days later. The repertoire of Vγ9JPC1 chains is essentially unchanged in the expanded subset compared to fresh cells (55), despite the fact that stimulation indices for individual clones vary 10–100-fold (56). Surprisingly, there were no significant differences in apoptosis after *in vitro* stimulation comparing Vδ2 cells from HIV-infected patients or uninfected controls (35) but the preferential loss of phosphoantigen responses in HIV disease still argues for a direct link between antigen specificity and depletion, apparently a different type of activation-induced cell death.

Gene expression array studies provided a surprising insight into infection and γδ T cell dysregulation that may impact Vδ2 cells. During HIV infection, mRNA for enzymes in the cholesterol biosynthesis pathway is upregulated (57, 58). Higher production of cholesterol is needed to meet the demands for viral membrane synthesis and more of the biosynthetic intermediates are required including prenyl pyrophosphates (phosphoantigens). With more phosphoantigen present, it is reasonable to expect greater activation of Vγ9Vδ2 T cells and this may explain the rapid decreases during acute infection when viremia is highest and the frequency of infected cells is also highest compared to other stages of disease.

**Figure 1:**
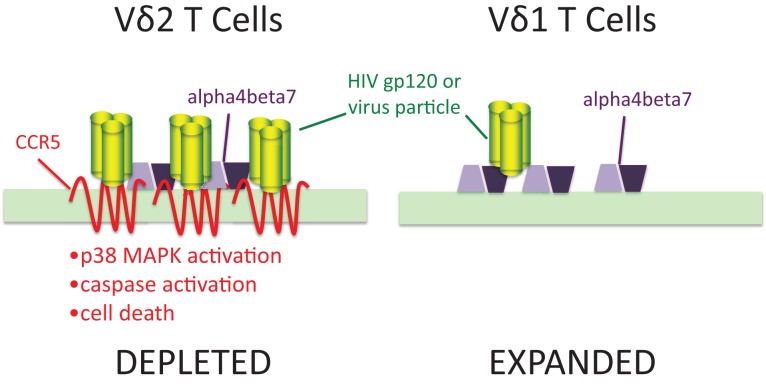
**Human immunodeficiency virus envelopes gp120 signaling through CCR5 is a mechanism for specific depletion of Vδ2 T cells**. Vδ2 T cells express high levels of two binding molecules for HIV gp120: chemokine receptor CCR5 and integrin α4β7. In Vδ1 cells, gp120 (green trimers) bind to α4β7 (purple polygons) but do not signal caspase activation or cause cell death. In Vδ2 T cells, the presence of 7-transmembrane CCR5 (red) binds gp120 and signals through its associated p38 MAP kinase to increase caspase activity and cell death. Adapted from Ref. (62,63).

We also know (49, 59) that a large proportion of activated γδ T cells express chemokine receptors including CCR5 and CXCR4 that are major co-receptors for HIV (Figure 1). These receptors bind sequences in the V3 loop of envelope glycoprotein gp120 and are important for viral entry. Both Vδ2 and Vδ1 cells express α4β7 integrin that also binds the V2 loop of gp120 (60, 61). CCR5 expression was only seen in the Vδ2 subset where it was estimated to be present at >50,000 molecules per cell surface (62), or roughly 10-fold higher surface density compared to activated αβ CD4 T cells. Treating expanded Vδ2 cells with gp120 lead to induction/activation of caspases followed by apoptosis. Inhibitor studies confirmed that signaling through CCR5 and its associated p38 MAP kinase, was necessary for this cell death pathway (62). We also detected gp120 binding to Vδ1 cells that could be blocked by ligands for α4β7 but was not affected by the CCR5 antagonistic drug Maraviroc. Flow cytometry showed that Vδ1 cells have no detectable surface CCR5 (63). The presence of high density CCR5 on Vδ2 T cells and its absence on Vδ1 T cells may explain specific cell killing of Vδ2 cells by gp120 that is similar to the pattern seen during HIV infection. Since high density CCR5 expression occurs on phosphoantigen-stimulated cells, this mechanism might account for the connection between antigen specificity and cell loss.

Several other models have been proposed to explain the γδ T cell defect in HIV disease. We first reported that Vγ9Vδ2 T cells activated *in vitro* were permissive for HIV infection (36) but the number of productively infected cells in patient samples seems to be very low. However, it was reported that human herpes virus 6 elevates CD4 on several cell types including γδ T cells (64) and could impact susceptibility of these cells *in vivo*. Both direct infection of intrathymic γδ T cell precursors (65) and inhibition of thymic development by HIV-infected γδ T cells (66) were proposed as models for specific or general T cell depletion. However, it is important to note that Vδ1 cells are not depleted during HIV disease as would be expected for a mechanism acting at the level of thymopoiesis. In addition, the normal human blood repertoire includes Vγ9Vδ2 cells that use both the JP and other rearrangements even though we emphasize Vγ9JP because of its responses to phosphoantigens. Sequencing studies showed that Vγ9Vδ2 cells using J segments other than JP, remained at normal levels and appeared to be unchanged during HIV disease (14). Further, the rapid depletion of Vγ9Vδ2 T cells before the onset of immunodeficiency and reactivation of pathogens like HHV-6, argues this is not a major mechanism for depletion. The impact of direct infection, despite infrequent CD4 expression or negative effects on thymopoiesis may contribute to Vγ9Vδ2 cell depletion but are not likely to be the major mechanisms.

Based on studies of former plasma donors in southern China who were infected at the same time and with similar strains of HIV, we know that Vδ2 levels correlate with viremia (hence levels of gp120) but not with CD4 T cell count (67). This adds support to a model where viral proteins are directly responsible for Vδ2 cell depletion as was proposed earlier (11). During natural history studies of HIV disease it will be important to identify patients being treated with CCR5 antagonists including Maraviroc, who may have increased Vδ2 T cells compared to patients treated with regimens that do not include this drug class.

**Figure 2:**
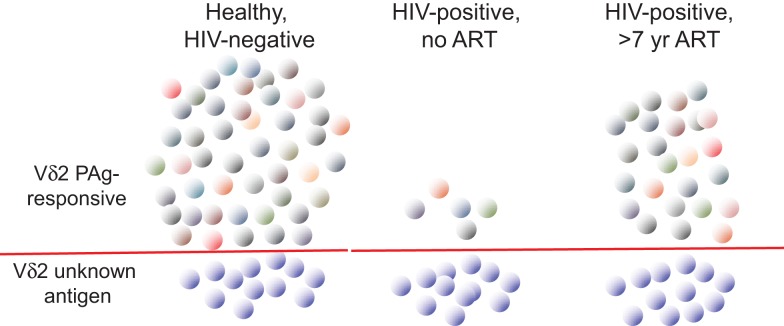
**The impact of HIV infection on circulating Vγ9Vδ2 T cells**. The characteristics of circulating Vγ9Vδ2 T cells (abbreviated Vδ2) are summarized for healthy, HIV-uninfected, and HIV-infected patients without antiretroviral therapy (ART) or after >7 years of ART. Individual cell clones are designated by colored symbols. Below the red line, we show the population of Vγ9Vδ2 T cells that do not respond to phosphoantigen (PAg) and remain at roughly equal numbers irrespective of infection or treatment. Without ART, HIV infection drives down the number and TCR complexity of PAg-responsive Vγ9Vδ2 (above the red line). After prolonged ART, TCR complexity recovers in the PAg-responding population (indicated by different colors) but the numbers and functions remain below uninfected controls. Adapted from Ref. (50, 70,74).

In order to understand better the dynamics of Vδ2 T cell depletion and the potential for immune reconstitution during therapy, extensive use was made of TCR repertoire analysis (Figure 2). The focus has been on the Vγ9 chain that is most tightly linked to phosphoantigen responsiveness due to the predominant Vγ9JP rearrangement (also called Vγ2Jγ1.2). Indeed, sequencing studies on the γ chain repertoire established the specificity of HIV-mediated deletion in the Vδ2 cell population (12) and subsequent work [reviewed in Ref. (68)] showed this TCR-specific defect is common to all HIV patients regardless of virus type, geographic distribution, race, or ethnicity. In the era before effective antiretroviral therapy, repertoire sequencing studies showed that patients with CD4 < 200 cells/mm^3^ had no detectable Vγ9JP cells in PBMC (14). When these patients initiated combination antiretroviral therapy and achieved virus suppression, we did not observe rapid rebound of Vδ2 cells in blood despite increases in CD4 cell count during 2–2.5 years of treatment. The lack of rapid Vδ2 cell recovery argues against the idea that depletion in peripheral blood is due to increased tissue compartmentalization, which is an important mechanism for controlling CD4 T cell count early in disease (69). A subsequent study of patients with longer intervals of antiretroviral therapy provided some initial evidence that Vγ9JP cells were reconstituted during virus suppression but the recovery rates were slow (13). More recently, studies with patients after >7 years of antiretroviral therapy revealed extensive reconstitution of the TCR cell repertoire (based on Vγ9 chain sequencing) and a population of Vγ9JP chains having nearly the complexity found in healthy, HIV-negative controls. Considering that some of the treated patients had nadir CD4 counts below 100 cells/mm^3^ and were expected to have no circulating Vγ9JP cells, reconstitution of this population was a surprising result (70).

The γδ T cell repertoire is defined by analyzing the number and frequency of clonotypes (predicted peptide sequences in the CDR3 or V-J region of the gamma chain) or nucleotypes (nucleotide sequences for these peptides) within a sample of the total population of TCR sequences in a blood or tissue specimen. These sequences are classified as public (identical amino acid sequences present in unrelated donors) or private (unique to one individual in our population of donors). For healthy, HIV-negative adults most of the clonotypes are public and many of the nucleotypes are also public indicating a high degree of similarity among unrelated individuals. This pattern is consistent with the use of monomorphic antigen-presenting molecules and is probably biased by nucleotide sequences in the germline regions of Vγ9 and JP that provide alternate routes to expressing the “germline” Vγ9JP rearrangement (70) using the mechanism of convergent recombination (71–73). In long-term treated HIV patients, there were clear differences between uninfected controls or treated patients who reconstituted the Vγ9 repertoire as measured by clonotype and nucleotype abundance (70). These differences prove that the repertoire was reconstituted by new cell synthesis and not by regrowth of a cell population that survived an initial HIV attack. Further, the reconstituted TCR repertoire included many sequences that should respond to phosphoantigen. Despite having a reconstituted repertoire and TCR against phosphoantigen, Vδ2 cells in these patients remained at levels well below matched, HIV-negative controls and had greater proportions of naïve cells indicating a lack of phosphoantigen responses and impaired positive selection *in vivo*. Patients also had lower expression of the CD56 cell surface marker (74) that is associated with cytotoxic effector function (75). Vδ2 cells from these long-term treated patients could be activated by potent *in vitro* stimulation whereupon they regained Fc receptor expression (CD16) and effector function in antibody-dependent cellular cytotoxicity (50). Despite reconstitution of the Vδ2 T cell population by new cell synthesis during prolonged ART, this subset does not recover to normal levels or function after treatment intervals of years to decades. The majority of Vδ2 cells found in treated HIV patients were generated and selected in an environment almost devoid of HIV due to effective therapy. The nature of this long-term defect is an obstacle to immune reconstitution and might contribute to chronic comorbid diseases in HIV patients.

A very small fraction of HIV patients actually have normal Vδ2 cell levels and function; these are elite controllers or natural virus suppressors defined as HIV patients with undetectable viremia except for occasional blips and no history of antiretroviral therapy (76). Among natural virus suppressor patients (approximately 0.5% of all persons with HIV infection), Vδ2 cell levels are equivalent to age, gender, and race-matched uninfected controls but there are significant differences in the Vγ9 chain repertoire (77). These repertoire differences reflect an early impact of HIV before the time when virus replication was controlled by host immunity and in this way, are similar to what we observed in treated patients where viremia was suppressed by chemotherapy. This also tells us that normal function of Vγ9Vδ2 T cells can return in HIV-infected patients but as yet, we have not uncovered the critical mechanisms for recovery that might help us to find new therapeutic targets.

## Milestone Achievements from the Middle Period of Studying γδ T Cells in HIV Disease

Nearly three decades of research produced many insights into HIV and γδ T cells. Studying HIV disease proved that Vγ9JPVδ2 cell depletion explained the defective response to phosphoantigen. Mechanisms were described for specific loss of Vγ9JPVδ2 cells that account for the relationship between depletion and phosphoantigen responsiveness. The findings showing that therapy reconstituted the Vγ9 repertoire but did not restore normal cell counts or function, were both encouraging and cautionary. Changes in γδ T cells are clearly related to HIV immune evasion but the long-term defects pose substantial challenges to the clinical management of HIV and efforts to reduce comorbid diseases.

## Problem of Durable γδ T Cell Damage Despite Chemotherapeutic Suppression of HIV

Natural history studies of HIV infection documented a brief, violent interval of acute infection characterized by extraordinarily high viremia and dramatic changes in immune cell populations, which continued until a stable viral set-point (78) was established. We prefer the term “immune set-point” that better describes the balance between immune system destruction and chronic infection that allows HIV to persist in an individual or in the human population. The immune set-point includes damage to Vδ2 T cell function that goes beyond what is seen in acute, adult malaria (79) for example, where there was a more limited transient loss of Vγ9Vδ2 T cells.

Both viral and immunological set-points designate the phase of disease when acute HIV infection gives way to a stable, persistent infection with slower progression and more time for virus to spread by sexual contact. In some virus examples, the acute to persistent transition reflects changes in viral gene regulation but in HIV disease the most important mechanisms are viral evasion of host immunity. Once persistence is established, chronic disease progression causes death around 11 years later in untreated HIV disease, as declining CD4 T cell counts and collapsing innate immunity increase the incidence and severity of lethal opportunistic infections, cancer, cardiovascular disease, and other terminal conditions. Evolutionary selection of HIV created a pathogen capable of spreading in the human population by relatively inefficient sexual transmission even when the median life expectancy after infection was only 11 years for adults and shorter for infected infants or elderly persons. Now, in the era of effective ART and much longer survival times, we are seeing a new scenario that was not part of the selection pressures driving natural evolution of HIV. Therapy with effective virus suppression repairs some of the earlier immune damage and likely reduces transmission rates (80) but key cell types including γδ T cells fail to recover normal numbers, phenotypes, or function, even after prolonged treatment that also fails to eradicate the virus. These enduring defects and their impacts on durable virus persistence or comorbidities of HIV are unanticipated – or better said – unselected consequences of HIV biology that are continuing decades after a viral immune evasion mechanism that caused damage to host immunity during the interval of acute infection.

Why these defects linger is a critical and unsolved question. We know that low numbers of viral RNA remain in plasma even during successful antiretroviral therapy (81) but these levels are around 10^5^ vRNA copies per ml of plasma lower that what is found during acute viremia. Accordingly, when the γ chain repertoire is reconstituted by new cell synthesis (70), we are dealing with cells that were birthed in an environment nearly devoid of HIV replication. Reconstitution is indeed slow as was predicted several years ago (13), but the return of function lags behind the gain of TCR repertoire complexity and as of now, there is no reliable estimate for the kinetics of functional recovery.

**Figure 3:**
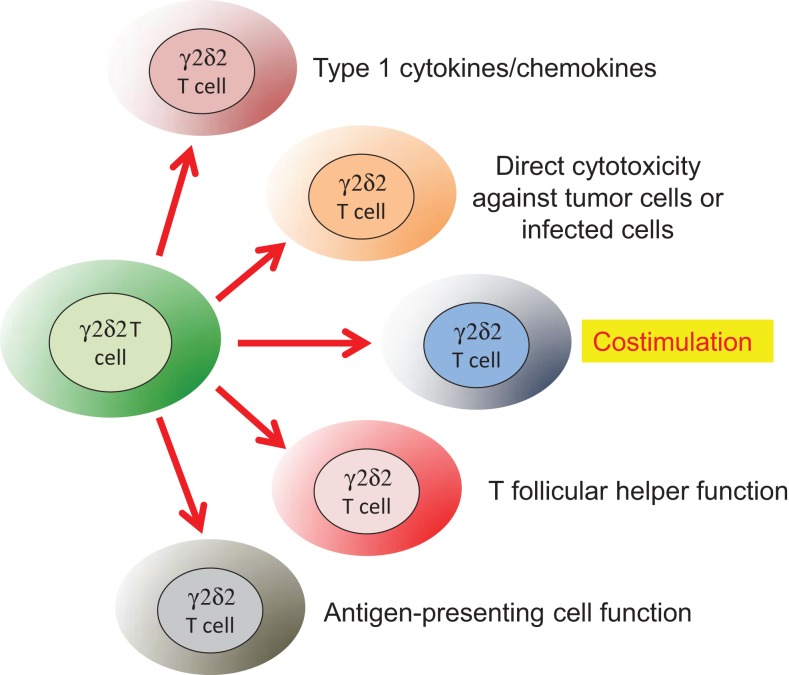
**Some of the known functions for human Vδ2 T cells that are related to HIV infection**. Naïve Vδ2 T cells can be stimulated to produce Type 1 cytokines/chemokines for antiviral immunity (47) or display potent lytic effect function against tumor cells (82, 83) or infected cells (47). Vδ2 T cells also provide and receive costimulation from both NK cells (86) and dendritic cells (87). They display some similarity to T follicular helper cells required for B cell responses (85) and can function as antigen-presenting cells (84).

The durable impact of HIV on γδ T cells impedes their contributions to key immune effector and regulatory mechanisms. Relating the list of possible γδ T cell functions to the known outcomes of HIV disease is a challenging undertaking and will trigger much debate. Our list of relevant Vδ2 T cell functions (Figure 3) includes control of antiviral immunity (47), tumor or infected cell killing (82, 83), antigen presentation (84), B helper T cell function (85), and costimulation of NK cells (86, 87) although alternate lists with additional functions can be imagined. In this review, we focus on the impact of losing Vδ2 T cell costimulation and why this loss can be an important underlying factor in HIV-associated comorbid diseases that are causing death and disability in persons despite effective viral suppression by ART.

The consequences of prolonged depression in Vδ2 T cell function may appear in unexpected comorbidities of HIV disease. For example, psoriasis affects 1–3% of patients with HIV (88), which seems paradoxical since this is a T cell-mediated autoimmune disorder (89). Normally, psoriasis is associated with influx of blood Vγ9Vδ2 T cells into the inflamed skin that may be a mechanism for resolving the acute condition. In HIV disease, we can imagine that the absence of Vγ9Vδ2 T cells or their chronically poor responses in patients treated with antiretroviral therapy will reduce their beneficial effect and increase the severity or duration of psoriasis in these patients. The Vγ9Vδ2 T cells are intimately related to both NK and dendritic cells through interactions that control cell activation and inflammation. Psoriasis is an example where the loss of Vγ9Vδ2 function removes a key protective mechanism.

**Figure 4:**
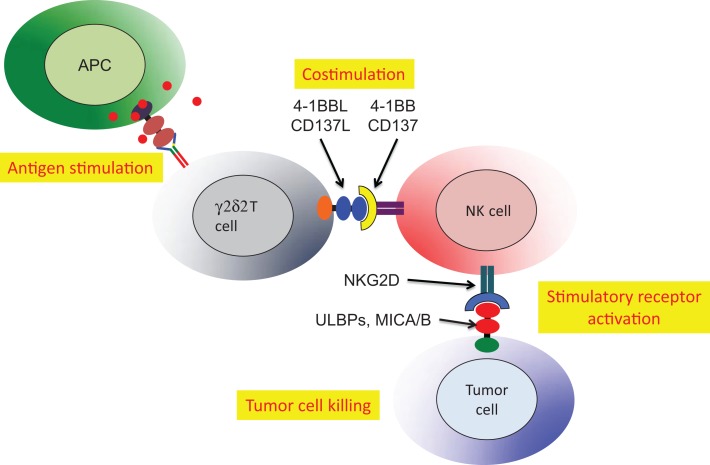
**Stimulated Vδ2 T cells co-stimulate NK to increase tumor cell cytolysis**. Activated Vδ2 T cells express 4-1BBL that binds 4-1BB on NK cells. The NKG2D receptor on NK is upregulated allowing stronger recognition of tumor cells expressing self-stress ligands ULBP or MIC A/B (86). HIV-mediated destruction of Vδ2 T cells will cripple the important mechanism for NK costimulation and reduce natural tumor surveillance.

Cell:cell interactions (cross-talk) involving γδ T cells were implicated in the activation of dendritic cells (90), enhancement of antibody production by γδ T cells with T follicular helper cell activity (85) and antigen presentation or cross presentation (84). An additional and more specific example of cross-talk involved NK tumor cytotoxicity, which was activated by Vδ2 T cells (86). The critical role for Vδ2 T cells in NK tumor cytotoxicity was mapped to expression of the 4-1BB ligand (CD137L). Phosphoantigen stimulation of human PBMC expanded the Vδ2 subset and upregulated both 4-1BB (costimulatory receptor) and NKG2D (activating receptor that recognizes stressed-self antigens) on the NK cells (Figure 4). A murine cell line expressing 4-1BBL could be substituted for Vδ2+ T cells indicating that this costimulatory ligand was the major signal for Vδ2 T cell enhancement of NK tumor cytotoxicity (86). This surprising result linked the anti-tumor effector activity of NK cells to the presence of phosphoantigens and activation of Vδ2 T cells.

**Figure 5:**
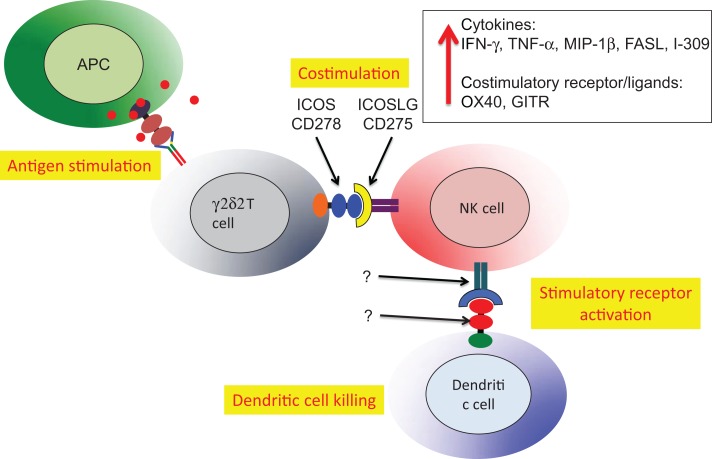
**Vδ2 costimulation of NK cells increases lytic effector activity against autologous dendritic cells**. The Vδ2 and NK cells interact through ICOS/ICOSL interactions resulting in upregulation of cytokine expression, increased mRNA for several other costimulatory receptors and increase lytic effector activity against dendritic cells (87). The relevant receptor(s) on NK and recognition molecules on dendritic cells have not been identified. Similar to the example in Figure 4, HIV-mediated depletion of Vδ2 is expected to reduce the capacity for NK killing of dendritic cells that will accumulate and promote chronic immune activation/inflammation that is a hallmark of HIV disease.

A second example of Vδ2 T cell costimulation of NK is enhancement of effector activity capable of eliminating immature or mature, antigen-presenting dendritic cells (Figure 5). Again, phosphoantigen-expanded Vδ2 T cells delivered a costimulatory signal to NK that increased the killing of autologous dendritic cells. Along with increased production of Type 1 cytokines and chemokines, the increased levels of ICOS on Vδ2 T cells binding to ICOSL on NK cells was related to improving cytotoxic killing of dendritic cells (87). The Vδ2 T cells are reciprocally activated by the cross-talk with NK and may also be effectors for dendritic cell killing, but these experiments are complicated by the use of anti-TCR or anti-CD3 antibodies to purify Vδ2 cells. When antibodies are used for positive selection, Vδ2 T cells are super-activated and the effects of costimulation are obscured. These studies on costimulation support an earlier model for NK:γδ:DC interactions that focused on γδ T cell cytokines that regulate NK and DC function (91). In this example, we see a key role for antigen-specific Vδ2 T cells in costimulation of NK cells that will then gain the capacity for killing activated dendritic cells and help to control chronic inflammation.

When Vδ2 T cell costimulation of NK cells is lost we might expect increased cancer risk among HIV patients, despite effective viral suppression by therapy. In HIV clinics of the University of Maryland, Baltimore, more than 15% of all HIV patients will have a cancer diagnosis and in 85% of those cases, cancer will be the cause of death (92). The great variety of cancers that increase in HIV patients suggests that a common mechanism of tumor control is missing or ineffective. Decreased tumor surveillance by NK cells may contribute to this comorbidity of HIV-associated cancer and the loss of Vδ2 T cell costimulation could be a key mechanism for the loss of effector function.

Vδ2 T cell depletion, loss of costimulation, and the consequent reduction in dendritic cell killing may have important effects on several HIV-associated comorbidities. Chronic immune activation with inflammation is a hallmark of HIV disease and a danger to patients even after virus replication is suppressed. The lingering defect in Vδ2 T cells and the linked failure to co-stimulate NK for dendritic cell killing, likely increases the risk for chronic activation/inflammation as potent antigen-presenting cells accumulate in the absence of normal control mechanisms. Indeed, Fauci’s group noted the lack of potent dendritic cell editing by NK in HIV-infected and treated patients (93) and defects in plasmacytoid dendritic cell–NK cross-talk in HIV patient specimens that reflected poor activation of NK during innate immune responses (94). Vδ2 T cells are also involved in reciprocal interactions with dendritic cells that affect activation or maturation of both cell types (90). Consequently, dysfunction of Vδ2 cells will affect NK and dendritic cell interactions as all three cell types are interdependent. When all three cell types are normal, there is a balance between cell activation and cell killing that modulates inflammation. When one piece is missing, in this case Vδ2 T cells, the control over inflammation is lost.

The examples for defective Vδ2 T cells in HIV-infected and treated patients have important parallels in the NK literature. There are many reports about the phenotype of circulating NK cells in patients where HIV is controlled by antiretroviral therapy. In these patients, NK have lower density of NKp46, NKp30, and NKp44 receptors (95) and often appear as CD56−/CD16+ cells lacking cytotoxic effector activity (96). Because HIV infection is associated with decreased NKG2D (activating) and increased NKG2A (inhibiting) receptor expression (97), even the increased levels of ULBP target ligands failed to trigger cytolysis of HIV-infected target cells. The earliest mark of NK dysfunction seems to be the decreased expression of Siglec-7 (98). When the lack of Siglec-7 is combined with lower expression of CD56 (99), we are beginning to define the phenotype of defective NK cells that is common among HIV patients. Whether these defects could be corrected by costimulation has not yet been tested. Clearly, there is a convergence of functional defects among Vδ2, NK, and dendritic cells in HIV and potent antiretroviral therapy does not restore the normal cell interactions or function of this regulatory triangle. It is worthwhile to look for defects in multiple mechanisms of immunity including those highlighted in Figure 3.

With highly effective antiretroviral therapy and increasing interest in eradicating HIV, it is important to look at the future of research on γδ T cells. Surely, Vδ2 T cells provide an attractive target for immunotherapy, probably using aminobisphosphonate compounds plus IL-2 or IL-15 to increase cell levels and functions. Patterned on a number of ongoing clinical studies in cancer (100–105), aminobisphosphonate drugs may be useful for correcting the HIV-associated Vδ2 T cell defect. A pilot study showed this approach was safe for HIV patients (106) but these studies have not been repeated or expanded to include clinical and immunological endpoints such as NK or dendritic cell phenotype and function. The lack of progress may be due to insufficient justification for the outcome of bisphosphonate/IL-2 therapy, since viremia can be suppressed more easily with once-a-day antiretroviral agents.

## Plea for Innovative Clinical Trials Targeting γδ T Cells in HIV Disease

Clinical research on therapeutic restoration of γδ T cells in HIV disease will require innovative clinical trial strategies. It may be difficult to launch therapeutic interventions such as aminobisphosphonate plus IL-2 for 2 or 3 months, then wait 20 years or more to accumulate significant data on changing rates of pulmonary arterial hypertension (found in <5% of HIV patients) or other comorbidities. While each of the comorbid conditions affects only a portion of the HIV population, their combined impact is substantial but clinical trials rarely lump disparate conditions into single endpoints. We might overcome this problem by using immunological endpoints that are related to chronic disease mechanisms. For example, direct immunotherapy targeting γδ T cells might be tested as a way to activate Vδ2 T cells that would normalize NK cell phenotype and function. Recovering of normal properties in two distinct lymphocyte subsets may be sufficient to document a clinical benefit, even though neither change is likely to result in short term changes to the incidence, prevalence, or severity of comorbid diseases.

## Summary

The Vγ9Vδ2 T cells in HIV-infected individuals contain the historical record of disease, documenting the intensity of initial depletion and reconstitution during therapy by their abundance, activity, and sequences of their TCR. They are barometers for current disease status, reflecting the capacity for resisting opportunistic infections, natural tumor surveillance, the control of immune activation/inflammation, and likely several other conditions critical to the clinical management of HIV disease. In addition, reconstitution of the TCR repertoire may be a measure for treatment success and help to identify patients with the highest potential response to immunotherapies targeted at Vδ2 T cell activation. Their compact TCR repertoire and extraordinarily high proportion of public Vγ9 chains facilitates comparisons among individuals or groups of patients. When combined with the sophisticated knowledge about cell surface marker expression including costimulatory receptors/ligands, and their patterns of cytokine/chemokine expression, these cells become critical tools for the immunology of infectious diseases. Adding to the interest is the availability of approved drugs that activate Vγ9Vδ2 T cells *in vivo* with high specificity, and a rich pipeline of compounds under development that may do even better. The missing link is a commitment to small interventional clinical trials testing the capacity for γδ T cell-targeted immunotherapies to alter the profile of immunity and recover normal control in diseases like HIV/AIDS.

## Conflict of Interest Statement

The authors declare that the research was conducted in the absence of any commercial or financial relationships that could be construed as a potential conflict of interest.
